# Effects of Pea (*Pisum sativum*) Prebiotics on Intestinal Iron-Related Proteins and Microbial Populations In Vivo (*Gallus gallus*)

**DOI:** 10.3390/nu16121856

**Published:** 2024-06-13

**Authors:** Abigail Armah, Cydney Jackson, Nikolai Kolba, Peter R. Gracey, Viral Shukla, Olga I. Padilla-Zakour, Tom Warkentin, Elad Tako

**Affiliations:** 1 Department of Food Science, Cornell University, Ithaca, NY 14850, USA; aaarmah22@students.desu.edu (A.A.); cdj53@cornell.edu (C.J.); nk598@cornell.edu (N.K.); prg8254@cornell.edu (P.R.G.); vs354@cornell.edu (V.S.); oip1@cornell.edu (O.I.P.-Z.); 2 Crop Development Centre, Department of Plant Sciences, University of Saskatchewan, 51 Campus Dr., Saskatoon, SK S7N 5A8, Canada; tom.warkentin@usask.ca

**Keywords:** iron bioavailability, pea seed coat, microbial population, brush boarder membrane, prebiotics, *Gallus gallus*, gut microbiome

## Abstract

Iron deficiency remains a public health challenge globally. Prebiotics have the potential to improve iron bioavailability by modulating intestinal bacterial population, increasing SCFA production, and stimulating expression of brush border membrane (BBM) iron transport proteins among iron-deficient populations. This study intended to investigate the potential effects of soluble extracts from the cotyledon and seed coat of three pea (*Pisum sativum*) varieties (CDC Striker, CDC Dakota, and CDC Meadow) on the expression of BBM iron-related proteins (DCYTB and DMT1) and populations of beneficial intestinal bacteria in vivo using the *Gallus gallus* model by oral gavage (one day old chicks) with 1 mL of 50 mg/mL pea soluble extract solutions. The seed coat treatment groups increased the relative abundance of *Bifidobacterium* compared to the cotyledon treatment groups, with CDC Dakota seed coat (dark brown pigmented) recording the highest relative abundance of *Bifidobacterium*. In contrast, CDC Striker Cotyledon (dark-green-pigmented) significantly increased the relative abundance of *Lactobacillus* (*p* < 0.05). Subsequently, the two dark-pigmented treatment groups (CDC Striker Cotyledon and CDC Dakota seed coats) recorded the highest expression of DCYTB. Our study suggests that soluble extracts from the pea seed coat and dark-pigmented pea cotyledon may improve iron bioavailability by affecting intestinal bacterial populations.

## 1. Introduction

Iron is an essential mineral in human nutrition and is necessary for life. The biological importance of iron arises from its involvement in essential metabolic functions as an intrinsic component in synthesizing oxygen transport proteins (hemoglobin and myoglobin) and cytochromes [1,2,3,4,5]. Its deficiency has been associated with consequences such as impaired cognitive and physical development, reduced productivity and fatigue, increased morbidity, and all-cause mortality, with potentially far-reaching effects on economic productivity [5,6,7,8,9,10]. Iron deficiency remains a major global public health challenge affecting nearly one-third of the world’s population, despite being one of the most abundant elements and its daily requirement being relatively low [5,11,12]. Despite several large-scale intervention programs, it is the most prevalent nutritional deficiency and the leading cause of anemia, disproportionately affecting women of reproductive age and children [1,5,7,13,14]. It is one of the most common micronutrient deficiencies in both developing and developed countries worldwide [14,15]. Although several factors contribute to the causes of iron deficiency, the leading nutritional cause in developing countries could be linked to low intake of iron-rich foods and poor bioavailability of dietary iron [1,14]. 

Pulses are an important source of micronutrients, including iron [13], and are potentially useful in alleviating micronutrient deficiency as they are commonly consumed in developing countries where iron deficiency prevalence is relatively high due to its low cost and nutritional value [16,17,18]. They contain non-heme iron, which generally has lower bioavailability than heme iron [18,19]. After being released from food, non-heme iron generally exists in the insoluble ferric form (Fe^3+^), which has low bioavailability and needs to be transformed into the ferrous form (Fe^2+^) prior to absorption [14,16,19]. This vital step impacts iron bioavailability and is enhanced by reducing agents such as organic acids and ascorbate in the gut lumen [19,20]. The absorption of non-heme iron occurs in the proximal portion of the duodenum where duodenal cytochrome b (DCYTB), a duodenal ferrireductase, reduces ferric iron (Fe^3+^) derived from food to the ferrous form (Fe^2+^) and is transported by the divalent metal transporter (DMT-1) across the apical brush border membrane into the labile iron pool within the cytosol of the enterocyte and then released to plasma by ferroportin-1 (Fpn1), a transmembrane carrier of divalent metals, expressed on the basolateral membrane of the enterocyte whose action is highly regulated by hepcidin [16,20,21]. 

Despite their rich nutritional value, the bioavailability of iron in plant-based foods including pulses has previously been reported to be low due to the presence of antinutritional compounds such as dietary fibers, phytic acid, and polyphenols in sufficient amounts, which interfere with the absorption and bioavailability of iron and other minerals [14,16,18,19]. Recent studies have, however, shown that some of these antinutritional factors (e.g., polyphenols and fibers) reported to reduce iron bioavailability have prebiotic properties and are metabolized by the gut microbiota to produce short-chain fatty acids (SCFA), which reduce the pH of the intestinal lumen, leading to the following: increased iron solubility and reduction of ferric iron (Fe^3+^) to its more bioavailable ferrous form (Fe^2+^), increased expression of mineral transport proteins in the epithelial cells, and subsequently, enhanced iron absorption [14,22,23,24,25,26,27,28]. These SCFA also trigger increased proliferation of epithelial stem cells, leading to increased absorptive surface area and hence increased iron absorption [22,29,30,31].

Peas (*Pisum sativum*) are one of the major pulse crops widely cultivated worldwide, including Canada, the United States, India, Russia, and China. They are rich in polysaccharides (including fiber and starch), proteins, micronutrients, and bioactive compounds such as polyphenolic compounds, which are unevenly distributed in various parts of the seed [32,33,34]. The pea seed consists of three distinct parts: the two cotyledons, seed coat, and embryonic axis representing about 89%, 10%, and 1%, respectively, of the whole seed [32,33]. The main storage substances, primarily proteins, and carbohydrates, are contained in the cotyledons. In contrast, the seed coat, a protective layer around the cotyledon, mainly consists of insoluble non-starch polysaccharides with negligible amounts of starch and contains the highest concentration of polyphenols [33,35,36]. Like other pulses, peas are an essential source of iron for human nutrition [37]. Although the seed coat makes up only about 10% of the whole seed, previous studies have reported the iron content in the seed coat of different pulses to be as high as 20% of the iron content in whole pulse seeds [38,39]. Apart from polyphenols, peas also contain other bioactive compounds, including the water-soluble raffinose group of oligosaccharides, which humans cannot digest due to the lack of essential enzymes needed to break them down [34,40]. Thus, intestinal bacterial populations decompose and metabolize these prebiotics, leading to elevated SCFA production, potentially improving iron bioavailability [34,40]. A recent in vivo study involving intra-amniotic administration of raffinose reported an increased relative abundance of probiotics, decreased pathogenic bacterial population, increased intestinal surface area, and improved iron bioavailability [41]. 

Pea seed coat removal (dehulling) is a primary procedure in producing split peas, ground pea flour, and other fractionated ingredients such as pea proteins [36,42]. Pea seed coat has been reported to have high amounts of polyphenols and other important prebiotic bioactive compounds [34,40,42]. Currently, the primary market for the hull of various pulses, including pea hull, is low-value animal feed and, rarely, human food such as meat products and high-fiber bread [36]. As a by-product and the mainstream waste of pea processing, large amounts of pea hull consisting of a mixture of pea seed coat, broken cotyledon, and embryonic axes is generated worldwide annually, creating tough disposal waste for millers; this is a major waste of an important prebiotic and polyphenol-rich by-product and a potential health-promoting food ingredient [36,43].

CDC Striker is a field pea cultivar with green cotyledons, white flowers, opaque seed coat, and medium-sized round smooth seeds. It was released by the Crop Development Centre, University of Saskatchewan, in 2002 and has good yield potential [44]. Similar to CDC Striker, CDC Meadow has good yielding potential with white flowers, opaque seed coat, and round, smooth seeds [45]. It has yellow cotyledons and was released by the Crop Development Centre, University of Saskatchewan in 2006 [45]. CDC Dakota, on the other hand, has yellow cotyledons, purple flowers, and a dun (tan) seed coat. It was released as a variety by the Crop Development Centre, University of Saskatchewan, in 2010. It is a high-yielding variety with grain yield similar to that of CDC Meadow and CDC Striker. This study aimed to investigate the potential effects of soluble extracts from the cotyledon and seed coat of three pea varieties (CDC Striker, CDC Meadow, and CDC Dakota) on the expression of brush border membrane iron-related proteins (DCYTB and DMT-1) and populations of beneficial intestinal bacteria in vivo using the *Gallus gallus* model.

## 2. Materials and Methods

### 2.1. Plants Materials—University of Saskatchewan Pea Varieties

#### Growing Conditions and Post-Harvest Handling

**Figure 1:**
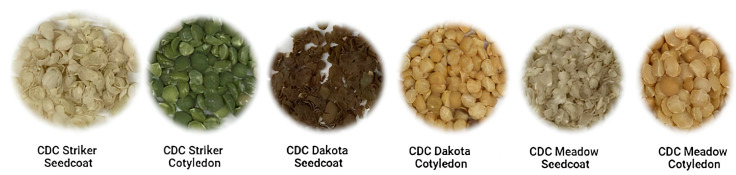
A photograph depicting pea seed coats and cotyledons used in assessing the iron bioavailability of three pea varieties.

Seed samples of CDC Striker, CDC Dakota, and CDC Meadow were provided by the Crop Development Centre, University of Saskatchewan (Figure 1). The varieties were grown in 2020 at Saskatoon, Saskatchewan, Canada. Best management practices for pea production in Saskatchewan were used. The varieties were harvested by plot combine; this was followed by the removal of straw and debris using screens and air. The seeds were subsequently stored at 17 °C in a seed storage building at the University of Saskatchewan until use for the current study. Pea samples were dehulled using a Satake dehuller (Satake Testing Mill Model TM05C(2)-T, Satake U.S.A. Inc., Stafford, TX, USA).

### 2.2. Extract Preparation

The pea cotyledon varieties to be tested were ground separately with a grinder, and 20 g of each variety of ground pea cotyledon and pea seed coat samples were suspended in 400 mL of 18 MΩ H_2_O and stirred continuously with a shaker for 60 min at 60 °C. The resulting extract was filtered to remove particulate matter and centrifuged at 1200× *g* for 10 min at 4 °C.

### 2.3. Polyphenols and Carbohydrate Analysis 

#### 2.3.1. Pea Cotyledon and Seed Coat Preparation

Samples were obtained through the use of absolute methanol with continuous agitation (stirring) in the dark for 2 h. Following this, the resulting mixture was centrifuged (10 min, 3000× *g*, Beckman Coulter, Allegra X-30R, Brea, CA, USA), and the supernatant was carefully decanted. Subsequently, the extract and washings were diluted in distilled water to achieve a 15% *w*/*v* concentration which was used immediately for further analysis as indicated below. Unused extract was stored at −20 °C. 

#### 2.3.2. Polyphenol Analysis 

Total polyphenol content (TPC) was assessed by following the method outlined by Waterhouse et al. [46]. In brief, the extract was mixed with the Folin–Coicalteu reagent and incubated for 40 min at room temperature. Twenty per cent (20%) sodium carbonate solution was then added to halt the reaction. Subsequently, the absorbance of the samples was measured at 765 nm using a UV–visible spectrophotometer (Thermo Fisher; Waltham, MA, USA). The total polyphenol content was quantified as gallic equivalents (GE) using a standard curve prepared under similar conditions. Gallic acid was solubilized in methanol and diluted to a linear range of 10, 50, 100, and 1000 µg.

The pH differential method [47] was used in assessing the total monomeric anthocyanin (MA) content of the extracts. In brief, the extracts were diluted with pH 1.0 (0.025 M potassium chloride) and pH 4.5 (0.4 M sodium acetate) buffers and incubated for 20 min at room temperature. Following this, the absorbance was measured at 520 nm and 700 nm using a UV–visible spectrophotometer. The equation below was used to quantify the total MA content as cyanidin-3-glucoside equivalents (CE).

((A520, pH1 − A700, pH1) − (A520, pH4.5 − A700, pH4.5)) × 529 × dilute factor × 1000/28,000

### 2.4. Study Design and Assessment

Day-old Cornish-cross broiler chicks (*n* = 42) were obtained from a commercial hatchery (Moyer’s Chicks, Quakertown, PA, USA) and randomly allocated to seven (7) treatment groups. All animal protocols were approved by Cornell University’s Institutional Animal Care and Use Committee (ethics approval code: 2020-0077). The seven treatment groups were as follows: Control (18 MΩ H_2_O only), 50 mg/mL CDC Striker cotyledon soluble extract, 50 mg/mL CDC Dakota cotyledon soluble extract, 50 mg/mL CDC Meadow cotyledon soluble extract, 50 mg/mL CDC Striker seed coat soluble extract, 50 mg/mL CDC Dakota seed coat soluble extract, and 50 mg/mL CDC Meadow seed coat soluble extract. The birds arrived in groups of ten. Birds from each group were randomly assigned to one of the seven treatment groups until all the birds had been assigned. The birds were randomly allocated to each treatment group (*n* = 7) as follows: Control (*n* = 7); CDC Striker cotyledon (*n* = 7); CDC Dakota cotyledon (*n* = 7); CDC Meadow cotyledon (*n* = 7); CDC Striker seed coat (*n* = 7); CDC Dakota seed coat (*n* = 7); and CDC Meadow seed coat (*n* = 7). Prior to gavage, the birds were allowed to fast for 24 h with ad libitum access to water. Birds allocated to the various treatment groups were gavaged with 1 mL of treatment solution. Post gavage, birds were allowed ad libitum access to water for 24 h. After 24 h, the birds were weighed by placing an empty weighing container on a digital scale and taring it to obtain only the weight of the birds. Each bird was gently placed in the weighing container and allowed to be gain stability prior to taking the weight measurements. All the birds were weighed at the same time of the day and subsequently euthanized by carbon dioxide (CO_2_) exposure. The duodenum and cecum sections were quickly removed from the carcass, immediately frozen in liquid nitrogen, and then stored at −20 °C until further analysis.

### 2.5. Isolation of Total RNA from Chicken Duodenum

Extraction of total RNA was carried out with 30 mg of the chicken proximal duodenal tissue samples (*n* = 7) using Qiagen RNeasy Mini Kit (RNeasy Mini Kit, Qiagen Inc., Valencia, CA, USA) based on the protocols outlined by the manufacturer. A rotor–stator homogenizer was used to briefly disrupt the tissue samples and homogenize them in buffer RLT containing β-Mercaptoethanol. The tissue lysate was centrifuged for 3 min at 8000× *g* using a microcentrifuge. The supernatant was transferred to another tube, combined with 600 µL of 70% ethanol, and mixed immediately. An amount of 700 µL of each sample was transferred to a RNeasy spin column and centrifuged for 15 s at 8000× *g*, and the flow-through material was discarded. The RNeasy spin columns were then transferred to new 2 mL collection tubes, and 500 µL of buffer RPE was pipetted onto each RNeasy column and centrifuged at 8000× *g* for 15 s. An additional 500 µL of buffer RPE was pipetted onto each RNeasy column and centrifuged for 2 min at 8000× *g*. Total RNA was eluted in 50 µL of RNase-free water and stored at −20 °C until use. All these steps were carried out under RNase-free conditions.

### 2.6. Real-Time Polymerase Chain Reaction (RT-PCR)

Using the extracted RNA, cDNA was created by a 20 μL reverse transcriptase (RT) reaction. This reaction was completed in a BioRad C1000 (Software 1844000, Hercules, CA, USA) touch thermocycler using the Improm-II Reverse Transcriptase Kit (Catalog #A1250; Promega, Madison, WI, USA). The cDNA concentration was obtained by Nanodrop (Thermo Fisher Scientific, Waltham, MA, USA) at an absorbance of 260 nm and 280 nm using an extinction coefficient of 33 (for single-stranded DNA). Genomic DNA contamination was assessed by a real-time RT-PCR assay for the reference gene samples. 

Real-Time Primer Design Tool Software (IDT DNA, Coralville, IA, USA) was used in designing the primers based on relevant gene sequences from the GeneBank database. The primer sequences relevant to non-heme iron metabolism used in this study are shown in Table 1, with the *Gallus gallus* primer 18S rRNA serving as the reference gene. Primer specificity was verified by BLAST searches against the genomic National Center for Biotechnology Information (NCBI) database.

### 2.7. RT-PCR Design

Using methods described previously, [48,49], cDNA (2 µL) was used for each 10 µL reaction together with 8 µL 2× BioRad SSO Advanced Universal SYBR Green Supermix (BioRad, Hercules, CA, USA), which included Taq DNA polymerase, buffer, SYBR green dye, and dNTPs. As was previously described [48,49], cDNA (2 µL) was used for each 10 µL reaction together with 8 µL 2× BioRad SSO Advanced Universal SYBR Green Supermix (BioRad, Hercules, CA, USA), which included Taq DNA polymerase, buffer, SYBR green dye, and dNTPs. Specific forward and reverse primers (Table 1) were added to each PCR reaction. Each run contained seven standard curve points in duplicate. A no-template control of nuclease-free water was included to detect and exclude DNA contamination in the PCR mix. Amplification of the double-stranded DNA was carried out in the Bio-Rad CFX96 Touch (Bio-Rad Laboratories, Hercules, CA, USA) under the following PCR conditions: initial denaturing at 95 °C for 30 s, 40 cycles of denaturing at 95 °C for 15 s, exposure to various annealing temperatures according to Integrated DNA Technologies (IDT) for 30 s, and elongating at 60 °C for 30 s. Data on gene expression levels were obtained as Cp values based on the automated method of the “second derivative maximum” as computed by the Bio-Rad CFX Maestro Software (Bio-Rad, Hercules, CA, USA). Reactions were run in duplicate for each of the genes of interest, and the results were quantified against a standard curve prepared by a 1:10 dilution. 

The software produced a graph of Cp against log 10 concentrations used, and the efficiencies were calculated as 10[1/slope]. A melting curve analysis (60–95 °C) was used to verify the specificity of the amplified real-time RT-PCR products after 40 cycles, resulting in several specific products, each with a specific melting temperature. The resulting PCR products were electrophoresed on a 2% agarose gel; the gel was stained with ethidium bromide and visualized under UV light. 

### 2.8. Microbial Sample and Intestinal Content DNA Isolation

Cecum samples (*n* = 7) were weighed, placed in sterile 15 mL tubes containing 9 mL PBS (pH 7.4) and homogenized by vortexing with sterile glass beads (4 mm diameter) for 3 min. Debris were removed by centrifuging at 1000× *g* for five minutes, and the supernatant was collected and centrifuged at 4000× *g* for 10 min. The pellets were washed with PBS and stored at −20 °C until DNA purification. The Wizard Genomic DNA purification kit was used according to protocols outlined by the manufacturer (Promega Corp., Madison, WI, USA).

### 2.9. Primer Design and PCR Amplification of Bacterial 16S rRNA

*Bifidobacterium* and *Lactobacillus* primers were used. The 16S rRNA was used as the universal primer and internal standard. The various bacterial groups were thus reported as relative proportions of the bacteria to the universal primer. The PCR products were applied to 2% agarose gel stained with ethidium bromide and quantified using Gel-Pro analyzer version 3.0 (Media Cybernetics LP, Rockville, MD, USA).

### 2.10. Statistical Analysis

The Statistical Package for Social Sciences (SPSS) version 27.0 software (IBM, Armonk, NY, USA) and R Studio (version 2022.07.2+576) were used for statistical analysis. Values were reported as mean values ± standard error mean (SEM). All the values were tested for normal distribution using the Shapiro–Wilk test. Once this assumption was confirmed, a one-way analysis of variance (ANOVA) was used in analyzing the data, followed by Duncan’s post hoc test to compare the differences between treatment groups. Results were considered to be statistically significant at *p* < 0.05.

## 3. Results

### 3.1. Polyphenol Analysis

The total polyphenolic content in the different pea cotyledon and seed coat samples are expressed as gallic acid equivalents (mg/g of sample, mean ± STD). From Table 2, CDC Dakota seed coats had the highest total polyphenolic content (0.826 mg GAE/g), and CDC Striker seed coats recorded the least (0.050 mg GAE/g) total polyphenolic content. No monomeric anthocyanins were detected in the pea samples analyzed.

### 3.2. Body Weight and Cecum-to-Body-Weight Ratio

From Table 3, the control (ddH_2_O) group had higher body weight (43.600 ± 1.364 g) compared to chicks in both cotyledon and seed coat treatment groups. This difference was statistically significant (*p* < 0.05) for the CDC Striker Cotyledon (38.857 ± 0.670 g), CDC Meadow Cotyledon (38.500 ± 2.320 g), and CDC Dakota seed coat (39.400 ± 0.980 g) groups.

There was no statistically significant difference (*p* > 0.05) in body weight for seed coat treatment groups compared to their corresponding cotyledon treatment groups for each of the pea varieties tested in this study (Table 3). The cecum-to-body-weight ratio was similar (*p* > 0.05) among all treatment groups when compared using ANOVA followed by Duncan’s test (Table 3).

### 3.3. Gene Expression of BBM Proteins

**Figure 2:**
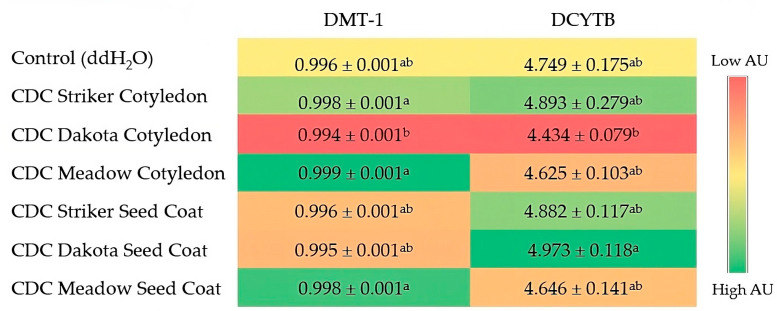
Heatmap showing the effect of soluble pea extract administration on intestinal gene expression of iron-related proteins. Values are the means (AU: arbitrary units) ± standard error mean (*n* = 7). Treatment groups not indicated by the same letter are significantly different (*p* < 0.05) as assessed by ANOVA followed by the Duncan post hoc test. DMT-1, Divalent metal transporter-1; DCYTB, duodenal cytochrome b.

Figure 2 (heatmap) shows the level of expression of intestinal iron-related proteins (DMT1 and DCYTB) involved in non-heme iron metabolism at the brush border membrane following the administration of soluble pea extracts, recorded in Arbitrary Units (AU). The rows in the heatmap represent the iron-related genes while the columns represent the samples obtained from the various treatment groups. The color intensity ranges from red (low expression) to green (high expression), indicating the relative levels of expression for each gene. There was no significant difference in the expression of DMT-1 and DCYTB between the control (ddH_2_O) group and the various cotyledon and seed coat treatment groups (*p* > 0.05). The relative expression of DCTYB was upregulated in the presence of seed coats for CDC Dakota and CDC Meadow compared to their respective cotyledons. This was statistically significant for the CDC Dakota treatment group (*p* < 0.05). There was no statistically significant difference in the regulation of DMT1 in the seed coat treatment groups compared to their corresponding cotyledon treatment groups for each of the dry pea varieties tested. 

Between the different color classes, DMT-1 expression was significantly reduced for the CDC Dakota cotyledon treatment group (*p* < 0.05) compared to the cotyledon treatment groups for CDC Striker and CDC Meadow.

### 3.4. Effects of Soluble Pea Extract on the Abundance of Intestinal Bacterial Populations

**Figure 3:**
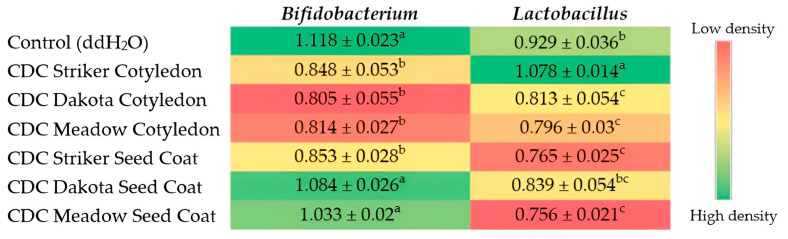
Heatmap showing genera-level bacteria population from cecal content of gavaged day-old chicks. Values are recorded as means (AU: arbitrary units) ± standard error means (*n* = 7). Treatment groups not indicated by the same letter are significantly different (*p* < 0.05) as assessed by ANOVA followed by the Duncan post hoc test.

Figure 3 (heatmap) displays the abundance of genera-level bacteria population from cecal content of gavaged day-old chicks. The genera-level bacteria are represented in the rows while the columns represent the treatment groups. The color scale ranges from red to green, where red indicates low density and green indicates high density. From our results, we observed a higher density of *Bifidobacterium* in the seedcoat treatment groups compared to the cotyledon treatment groups.

The relative abundance of *Bifidobacterium* as determined by 16S rRNA gene sequencing was higher in all three seed coat treatment groups compared to the cotyledon treatment group for each of the three dry pea varieties tested. This difference was statistically significant (*p* < 0.05) only for CDC Dakota and CDC Meadow. The relative abundance of *Lactobacillus* spp., on the other hand, was significantly higher (*p* < 0.05) in the CDC Striker cotyledon treatment group compared to the CDC Striker seed coat treatment group (Figure 3). The difference in abundance of the *Lactobacillus* population was statistically insignificant (*p* > 0.05) for the other pea varieties tested.

The relative abundance of *Bifidobacterium* in the Control (ddH_2_O) group was significantly higher compared to all three cotyledon treatment groups and the CDC Striker seed coat group. With regards to *Lactobacillus* spp., except for the CDC Striker Cotyledon and CDC Dakota seed coat, the Control (ddH_2_O) group had a significantly higher relative abundance of *Lactobacillus* spp. compared to all other treatment groups.

## 4. Discussion

In this study, the potential effects of soluble extracts from the cotyledon and seed coats of different pea varieties on the expression of brush border membrane iron-related proteins (DCTYB and DMT-1) and populations of beneficial intestinal bacteria was assessed using the *Gallus gallus* model. The *Gallus gallus* model in previous studies has been proven to be valid for initial in vivo screening of iron bioavailability in staple foods due to its sensitivity to micronutrient deficiency, taxonomic similarities at the phylum level between humans and this model, as well as genetic similarities in nutrient metabolism proteins between the two [34,41,48,50,51,52,53].

Oral gavage feeding has been used in previous studies [25,54,55,56,57] to deliver test compounds and nutrients to animals due to its ability to deliver precise and accurate volumes of test compounds [58,59] and has been used to investigate iron absorption and expression of iron related proteins including DMT1 in rats shortly (<24 h) after oral gavage [54,55]. Additionally, several studies have shown an increase in iron absorption [60,61,62] and decrease in DMT1 [62] in *Gallus gallus* (broilers) as early as 30 min after the perfusion of intestinal segments with solutions containing iron.

There were no statistically significant differences in the body weight of broiler chicks in the various treatment groups. While the control group (ddH_2_O) recorded a slightly higher mean body weight compared to some treatment groups, this may not necessarily be attributed to the treatment, given that only one-time bodyweight measurements were obtained. Despite the birds having been randomly assigned to the different groups, it is unlikely that each group would have precisely the same mean body weight. This is because even with randomization, there may still be differences due to chance [63,64] where some groups might have significantly different weight compared to others. Additionally, the body weight of the birds was obtained after randomization, and this may have contributed to the control group having a higher mean body weight compared to the treatment groups.

With respect to gene expression of iron metabolism proteins, even though no significant difference was recorded between the control and treatment groups, DCYTB was upregulated for the seed coat treatment groups in two of the pea varieties tested in the present study (CDC Dakota and CDC Meadow) compared to their corresponding cotyledon treatment groups. This difference was statistically significant for CDC Dakota, a pea variety with a dark-brown-pigmented seed coat with relatively higher total polyphenol content in its seed coat (Table 2). DCYTB is a brush border ferrireductase protein on the apical membrane of the intestinal enterocyte which reduces ferric iron (Fe^3+^) to the more soluble and readily absorbable ferrous (Fe^2+^) form which is then imported into the cytoplasm by divalent metal transporter-1 (DMT-1) [65,66]. Thus, the upregulation of DCYTB in the seed coat treatment groups for CDC Dakota and CDC Meadow compared to their corresponding cotyledon treatment groups indicates more ferric iron (Fe^3+^) was being converted to the soluble, more readily absorbable ferrous (Fe^2+^) form in the seed coat treatment groups. Legumes such as peas are good sources of ferritin, mostly found in the cotyledon due to iron remobilization from the nodules and leaves during seed maturation [67,68,69]. According to Dahl et al. [40], pea seed coat is mainly made up of water-insoluble polysaccharides, specifically cellulose, while the cotyledon contains polysaccharides with varying degrees of solubility, including pectin, hemicellulose, and cellulose [36,40]. An in vitro study by [70] reported that pectin, an anionic heteropolysaccharide polymer [71] had high iron binding capacity, which led to the formation of unabsorbable complexes with dietary iron, while cellulose bound none [14,70]. Similar findings were reported by Bosscher et al. [72]. This, to some extent, could explain the decreased expression of DCYTB in the cotyledon treatment groups, as some iron in the pea cotyledon may be bound to pectin and, therefore, unavailable for absorption. 

Pea seed coats are predominantly composed of insoluble non-starch polysaccharides with negligible amounts of starch [35,36] while the cotyledon comprises fiber, carbohydrates, proteins, minerals, and meager amounts of lipids [35]. Previous studies have shown some intestinal bacteria to reduce intestinal pH through the fermentation of prebiotics [73,74] which allows ferric iron to be converted to the reduced and more soluble ferrous form, making it readily available for absorption [75]. This may have occurred in our study as the *Bifidobacterium* population was increased in the seed coat treatment group. Non-digestible food ingredients have the ability to modulate the gut microbiota as both the *Gallus gallus* model and humans alike lack the necessary enzymes to digest most of these complex carbohydrates and polysaccharides, which are metabolized by microbes in the gastrointestinal tract to generate SCFAs, including butyrate, acetate, and propionate which are responsible for decreased pH in the intestinal lumen and thus increases iron solubility, making it more readily absorbable [76,77,78,79].

Though some plant-derived polyphenols are known to bind non-heme iron and impede its bioavailability [75], some studies have identified polyphenols that can reduce ferric iron to the ferrous form and promote iron absorption [80,81]. Catechin, 3,4-dihydroxybenzoic acid, kaempferol, and kaempferol 3-glucoside in black beans were reported to promote iron absorption in vitro [80]. Polyphenols are known to be concentrated in the seed coat [30,57,58], with pigmented seeds containing higher concentrations of polyphenols than non-pigmented ones [34,82]. The total polyphenol analysis in this study revealed that CDC Dakota, having a dark-brown-pigmented seed coat, had significantly higher total polyphenolic content (0.826 mg GAE/g) compared to the non-pigmented seed coat varieties (0.050 mg GAE/g and 0.069 mg GAE/g, respectively, for the CDC Striker seed coat and CDC Meadow seed coat). Moreover, a recent study reported significantly higher concentrations of iron-promoting polyphenols such as 3,4-dihydroxybenzoic acid (12.77 μg g^−1^ dry wt; *p* < 0.001), catechin (0.06 ± 0.01 μg g^−1^ dry wt; *p* < 0.001), and epicatechin (0.43 ± 0.15 μg g^−1^ dry wt; *p* < 0.001) in purple flowers and dun seed pea varieties including CDC Dakota compared to their concentrations in non-pigmented pea varieties (1.68 ± 0.84 μg g^−1^ dry wt; 0.03 ± 0.00 μg g^−1^ dry wt; and 0.03 ± 0.00 μg g^−1^ dry wt for 3,4-dihydroxybenzoic acid, catechin, and epicatechin, respectively) [83]. Since these polyphenols promote iron absorption [80], it is not surprising that this study recorded a statistically significant increase in the expression of DCYTB for the dark-brown-pigmented seed coat treatment group CDC Dakota compared to its cotyledon treatment group (*p* < 0.05), while also recording the highest value for the expression of DCYTB compared to the other non-pigmented seed coat treatment groups even though this was not statistically significant. Along the same line, the dark-green-pigmented cotyledon treatment group, CDC Striker, also recorded the highest expression of DCYTB compared to the other cotyledon treatment groups, even though this was statistically insignificant. Pea varieties with white seed coats tested in this study (CDC Striker and CDC Meadow) recorded no significant difference in the expression of DCYTB between their respective seed coat and cotyledon treatment groups. 

The current study recorded no significant difference in the expression of DMT-1 for the three pea varieties tested compared to the water control (*p* > 0.05). There was also no significant difference in DMT-1 expression between the seed coat and cotyledon treatment groups. Iron absorption, to some extent, is regulated by intracellular iron concentration in the enterocytes, and at the transcriptional level, the expression of DMT-1 is induced by iron deficiency to upregulate iron absorption [51,84]. Since the body lacks an active iron excretion system, iron homeostasis is tightly regulated to maintain iron balance, and this is predominantly controlled at the level of iron absorption from the intestines [81,85]. Thus, in a state of iron deficiency, more iron is mobilized from body iron stores and increased intestinal iron absorption [50]. These processes are, however, downregulated when the body is iron-replete [75]. It is therefore plausible that in this study, even though DCYTB was upregulated for the seed coat treatment groups, making more reduced and soluble iron (Fe^2+^) available for absorption, no significant increase in DMT-1 expressions was observed as the animal models used in this study were not iron deficient. This agrees with findings from previous studies, which reported higher expressions of both DMT-1 and DCYTB in iron-deficient broiler chickens with increased cellular iron uptake relative to iron-adequate ones [51]. 

The gut microbiome is one of the most densely populated communities, consisting of about 150 times more genes compared to the human genome [29]. Like humans and most other animals, the *Gallus gallus* model has a complex and dynamic gut microbiota [86] which is greatly influenced by diet, environment, and host genetics [48,53,87]. Its gut microbiota shares considerable similarities with humans at the phylum level, with *Actinobacteria*, *Proteobacteria*, *Bacteroidetes*, and *Firmicutes* representing the four most abundant bacterial phyla in both [48,50,53,88]. The genus *Bifidobacterium* consists of gram-positive bacteria belonging to the phylum *Actinobacteria* and represents one of the most dominant microbial populations in the gut of various animals [89,90].

Similar to findings from previous studies which reported increased abundance of *Bifidobacterium* resulting from consumption of various prebiotics—inulin [91,92] and galactooligosaccharides [78,79]—relative abundance of *Bifidobacterium* in this study was significantly higher in the seed coat treatment groups for two of the pea varieties tested in this study (CDC Dakota and CDC Meadow) compared to their respective cotyledon treatment groups. Peas contain galactooligosaccharides, and the seed coat mainly contains water-insoluble polysaccharides, which have beneficial prebiotic effects [36,40]. They also contain various polyphenols, which exert beneficial effects as prebiotic substrates due to their low bioavailability, allowing them to reach the colon unaltered [23,40]. This prebiotic characteristic of polyphenols leads to an increase in the growth and settlement of probiotic bacterial families such as *Bifidobacteriacece*, which can decompose and metabolize polyphenols while inhibiting pathogenic bacterial growth [23,40,93] by altering their cell wall stiffness and the permeability of the cell membranes of pathogenic bacterial species, leading to leakage of intracellular substances and irreversible damage which inhibits their cell growth and reproduction [23,94,95]. Pea seed coats have been reported to contain the highest concentration of polyphenols, especially in dark-seeded varieties [34,39,40,82]. In this study, CDC Dakota seed coat, a dark-brown-pigmented seed coat recorded the highest phenolic content (0.826 mg GAE/g) in comparison to its cotyledon and other non-pigmented seed coats. Thus, it is unsurprising that the relative abundance of *Bifidobacterium* was higher in the seed coat treatment groups compared to the cotyledon treatment groups, with the CDC Dakota seed coat treatment group (which is dark-brown-pigmented) recording the highest abundance of *Bifidobacterium*. Furthermore, *Bifidobacterium* is considered an essential component of a healthy microbiome due to its involvement in the production of SCFAs which carry out important physiological functions as immunomodulators and metabolic regulators and decrease the growth of potential pathogenic intestinal microbes [78,96]. 

Consistent with findings from this study, refs. [24,25] reported an increased beneficial bacterial population from the consumption of prebiotics which resulted in increased SCFA production. These SCFAs play an essential role as a significant energy source for both colonic epithelial cells and the gut microbiota while also promoting microbial growth and preventing colonization by pathogenic microbes by reducing space and nutrients available [29,78]. Recent studies have demonstrated the role of SCFAs in promoting the proliferation of intestinal stem cells [30] which are essential for maintaining homeostasis in the intestinal epithelium [31], enhancing the absorptive surface area of the enterocyte, and promoting iron absorption [29].

Through its action on maintaining intestinal epithelium cell homeostasis, increased SCFA production has been shown to strengthen intestinal barrier integrity, reduce inflammation, and reduce circulating hepcidin levels [29]. This is particularly important for improving iron bioavailability as hepcidin is considered to be the “master” regulator of iron homeostasis and plays a critical role in the regulation of body iron intake due to its role as a negative iron regulator which usually inhibits iron absorption [75,97]. Moreover, a study by [84] reported that an acute increase in hepcidin concentration induced a decrease in DMT1 expression and reduced iron absorption in vitro using human polarized intestinal cells and ex vivo using mouse duodenal segments. Thus, an increased population of SCFA-producing bacteria such as *Bifidobacterium* is crucial for maintaining intestinal barrier integrity and improving non-heme iron bioavailability.

Apart from improving the abundance of beneficial SCFA-producing bacteria in the guts, pea prebiotics have been demonstrated to be particularly important as they contain naturally occurring oligosaccharides (non-fructosylated α-galactooligosaccharides (α-GOS)) with reduced side effects on gas production from colonic bacterial fermentation, an issue of concern with several other legume seeds [78]. Previous studies by [98] indicated that no adverse outcomes were reported when pea seed coat fiber was added to inulin in snacks and consumed by children with constipation while bowel movement frequency was improved. Thus, dietary consumption of pea seed coats could potentially provide beneficial effects in improving intestinal health. Furthermore, other studies have discussed the incorporation of pea seed coat as a fiber source in rice-based snacks, chicken nuggets, wheat bread, as well as other baked goods [36,99,100]. This could potentially enhance the iron nutriture of these products. Besciani et al. [100] reported that rice-based snacks with 15% and 30% pea seed coat were more acceptable to consumers compared to snacks made with 100% rice.

## 5. Conclusions

In conclusion, the current preliminary study demonstrates that pea seed coat has the potential to modulate intestinal health and improve iron absorption through its bifidogenic effects on the gut microbiota. Further studies on the polyphenolic profile of dark-pigmented pea seed coats and their effects on SCFA production and iron absorption as well as additional studies investigating the expression of additional iron-related proteins are warranted to confirm this. Using iron-deficient animal models for subsequent studies would further demonstrate the effects on DMT-1 expression and other iron-related proteins.

## Figures and Tables

**Table 1:** DNA primer sequence used in this study.

Analyte	Forward Primer (5′-3′)	Reverse Primer (5′-3′)	Base Pair	GI Identifier
DCYTB	CATGTCATTCTCTTCCAAAGTC	CTCCTTGGTGACCGCATTAT	103	20380692
DMT-1	TTGATCAGAGCCTCCCATTAG	GCGAGGAGTAGGCTTGTATTT	101	206597489
18S rRNA	GCAAGACGAACTAAAGCGAAAG	TCGGAACTACGACGGTATCT	100	7262899

DMT-1, Divalent Metal Transporter–1; DCYTB, Duodenal cytochrome B; 18S rRNA, 18S Ribosomal subunit RNA.

**Table 2:** Total polyphenol content (TPC) and monomeric anthocyanins (MA) were estimated in the different pea cotyledons and seed coats as appropriate.

Pea Type	TPC (mg GAE/g)	MA (CE/g)
CDC Striker Cotyledon (2020 SPG)	0.106 ± 0.001	ND
CDC Dakota Cotyledon (2020 SPG)	0.143 ± 0.001	ND
CDC Meadow Cotyledon (2020 SPG)	0.131 ± 0.003	ND
CDC Striker Seed Coat (2020 SPG)	0.050 ± 0.002	<1/ND
CDC Dakota Seed Coat (2020 SPG)	0.826 ± 0.005	ND
CDC Meadow Seed Coat (2020 SPG)	0.069 ± 0.001	ND

ND: Not detected; Values are shown in mean ± stand error mean.

**Table 3:** Mean body weight and cecum-to-bodyweight ratio of broiler chicks used in this study.

Pea Type	Body Weight (g)	Cecum-to-Bodyweight Ratio (%)
Control (ddH_2_O)	43.6 ± 1.4 ^a^	0.014 ± 0.003 ^a^
CDC Striker Cotyledon (2020 SPG)	38.8 ± 0.7 ^b^	0.014 ± 0.002 ^a^
CDC Dakota Cotyledon (2020 SPG)	39.8 ± 0.9 ^ab^	0.011 ± 0.001 ^a^
CDC Meadow Cotyledon (2020 SPG)	38.5 ± 2.3 ^b^	0.014 ± 0.001 ^a^
CDC Striker Seed Coat (2020 SPG)	41.6 ± 1.5 ^ab^	0.010 ± 0.001 ^a^
CDC Dakota Seed Coat (2020 SPG)	39.4 ± 0.9 ^b^	0.013 ± 0.001 ^a^
CDC Meadow Seed Coat (2020 SPG)	40.7 ± 0.9 ^ab^	0.014 ± 0.001 ^a^

Treatment groups marked with different letter are significantly different (*p* < 0.05) as assessed by ANOVA followed by the Duncan post hoc test. Values are shown in mean ± stand error mean (*n* = 7).

## Data Availability

The original contributions presented in the study are included in the article further inquiries can be directed to the corresponding author.
